# The Clergy and Doctors

**Published:** 1889-01

**Authors:** 


### THE CLERGY AND THE DOCTORS.

While it is true that a comparison should have a parallel signifficance, yet due consideration should be given to the characteristic features which predominate in the one over the other; for there is always more or less superiority in one of the two of comparable objects or theories. This is especially true in contrasting nature with art, and divine things with those purely temporal.

The writer has lately seen some discussions in regard to the preaching of the Gospel and the Medical profession, and where some of the latter fraternity have taken the position, (negatively) at least, that the profession of medicine is superior to the other, as some have expressed it, that the doctor is a greater man than the preacher. As I have been requested by some friends to give my views upon the question, I do so through the medium of the RECORD in the fraternal spirit with which I hope they will be received.

To sustain the proposition that the doctor of Medicine is a greater man than the minister of the Gospel, one must prove that the preservation of physical vigor is of more value than mental and moral health; and that a life on earth with its short duration, and with all the cares, sorrows, and incomplete fulfilments which are the common heritage of mankind, is of more priceless worth than eternal life of endless joy and immortal youth and health.

No one can sustain such a position as this, for it is contrary to the laws of sociology and of moral and mental philosophy. Those branches of science teach that the most important of all education, and the first to be taught, is the cultivation of the heart.

No one will deny that this is not taught, and cannot be, only in a christian country, and that by the co-operation of such teachings in the word of God through a christian press, and from ihe pulpit by the accredited ministers of the Gospel.

The profession of Medicine justly and frequently has been awarded the second place to the christian ministry, but it ought not, and cannot take the precedence. The true consecrated ministers of the Gospel are the evangels of fallen humanity, whom the Great Father of us all has sealed and set apart for the salvation of immortal souls. It is not only their high and holy mission to offer to the thirsty lips of sinful man the all-healing waters of eternal life, but like the master they serve, to go about doing good, and to pour heavenly balm into sorrowful hearts, to listen with ready, Christ-like sympathy to the tale of sorrow, perplexity, and trial, and to be counsellors and peace-makers to friends at variance, who perhaps only need a word fitly spoken, and the helping hand of a brother to forgive and forget, and be to each other as before.

The ideal doctor of Medicine also, is not only able under God to successfully minister to the physical health of man but at the same time can point him to the great Physician of the sin-sick soul.

For this reason, I have often thought that the tenets and the practice of christianity were peculiarly adapted to the profession of medicine, and as I often say to my class, I cannot see how it is possible for one to study and practice medicine, and not be a devout believer in the existence of a gracious and omnipotent God.

No two professions, by their nature and purposes go more fittingly side by side, than the ministry of the Gospel and ministry of Medicine, nevertheless, the latter never has had, and never can mantain supremacy over the other, because the mission of the Gospel (not the man) is as superior to that of medicine as mind over matter, the incorruptible over the corruptible, and eternity to a little span of time on this earth, which is destined to be blotted out of existence, while the immortal spirit of man will live through the endless ages.

One could enlarge here upon the respective obligations of the ideal minister and the ideal doctor, did space permit, but it is hoped that we all, especially our students of medicine, will enter upon the new year with an exalted purpose and fixed resolution to honor and to do our whole duty in the humane and sacred mission we undertake when we become doctors of medicine.

